# mTOR Signaling in the Regulation of CD4+ T Cell Subsets in Periodontal Diseases

**DOI:** 10.3389/fimmu.2022.827461

**Published:** 2022-02-10

**Authors:** Qian Jiang, Xiaobin Huang, Wenjing Yu, Ranran Huang, Xuefeng Zhao, Chider Chen

**Affiliations:** ^1^ Department of Oral and Maxillofacial Surgery and Pharmacology, School of Dental Medicine, University of Pennsylvania, Philadelphia, PA, United States; ^2^ Department of Orthodontics, School of Dental Medicine, University of Pennsylvania, Philadelphia, PA, United States; ^3^ State Key Laboratory of Oral Diseases, National Clinical Research Center for Oral Diseases, West China Hospital of Stomatology, Sichuan University, Chengdu, China; ^4^ Center of Innovation and Precision Dentistry, School of Dental Medicine, School of Engineering and Applied Sciences, University of Pennsylvania, Philadelphia, PA, United States

**Keywords:** periodontal disease, inflammatory, CD4^+^ T cells, immune response, mTOR signaling

## Abstract

Periodontal disease results from the inflammatory infiltration by the microbial community which is marked through tooth mobility and alveolar bone resorption. The inflammation in periodontal disease is mediated by CD4^+^ T cells through cytokine secretion and osteoclastogenetic activity. Historically, the inflammatory model in periodontal disease is described through disruption of the balance between two subsets of T helper cells which are T-helper type 1 (Th1) and T-helper type 2 (Th2). However, more and more studies have found that apart from subsets of helper T cells, regulatory T-cells and Th17 cells are also involved in the pathogenesis of periodontal diseases. Growing evidence proves that helper T cells differentiation, activation, and subset determination are under the strong impact of mTOR signaling. mTOR signaling could promote Th1 and Th17 cell differentiation and inhibit Treg commitment through different mTOR complexes, therefore we anticipate a regulation effect of mTOR signaling on periodontal diseases by regulating CD4^+^ T cell subsets. This review aims to integrate the topical researches about the role of different types of Th cells in the pathogenesis of periodontal diseases, as well as the regulation of mTOR signaling in the specification and selection of Th cell commitment.

## Introduction

A new periodontitis classification scheme has been adopted by the World Workshop in 2018, in which three forms of periodontitis can be identified: necrotizing periodontitis, periodontitis as a manifestation of systemic disease, and the disease previously recognized as “chronic” or “aggressive” are now grouped under a single category, “periodontitis” and are further classified based on a multi-dimensional staging and grading system (1, 2). Periodontitis is an inflammatory disease that damages the soft tissue and, without treatment, can result in progressive destruction of the periodontal ligament and alveolar bone with periodontal pocket formation, gingival recession, and even tooth loss (3). Periodontal disease does not only affect the gums tissue but also has been stated to be a potential risk factor for systemic diseases such as cardiovascular disorders, low birth weight infants, and several others due to exposure of bacteria from periodontal tissue to blood and the resultant production of inflammatory mediators (4–6). The periodontal disease has been better understood through the latest findings in the field of microbiology and immunology. Recent researches in microbiology have shed better light on the mechanism behind the development of periodontitis (7), whereas immunobiology studies have revealed that periodontitis is caused by immune responses against bacterial infection which eventually results in periodontal tissue damage (8). It has been established that the immune responses of the human immune system determine the susceptibility to periodontitis. However, the exact role of different subsets of immune cells in periodontitis, the role of immunity in alveolar bone destruction, and the specific signaling pathways involved in immune regulation in periodontitis remain unclear.

Periodontitis is the inflammatory process with a dense accumulation of immune cells, including neutrophils, macrophages, lymphocytes, plasma cells, and mast cells (9). The lesion is initiated by the response of resident leukocytes and endothelial cells to the bacteria, which produce cytokines and cause vasodilatation of local blood vessels. Neutrophils migrate out of blood vessels into the site of infection in response to chemokines. The numbers of neutrophils increase and macrophages, lymphocytes, plasma cells, and mast cells also appear in the connective tissue. The following stage is the established lesion, which is the period of transition from the innate immune response to the acquired immune response. At this stage, plasma cells, macrophages, and T and B lymphocytes are dominant. The final stage is the advanced lesion, which is characterized by irreversible attachment loss and bone loss (10). T cells play an important role in this process of the immune response, regulating the polyclonal activation of the B cells (11), inducing osteoclastogenesis *via* RANKL activation, and producing several periodontitis-associated cytokines, such as IFN-γ, TNF-α, and interleukin (IL)-17 (12, 13).

It has been reported that human CD4^+^ T cells are involved in the immune response against oral microorganisms in periodontal diseases (14). In general, CD4^+^ T cells with regulatory function play a crucial role in controlling the immune responses during host defense (15). Different subsets of CD4^+^ T play role in the chronic inflammatory conditions distinctively (16), however, their exact contribution towards the destruction of the periodontal tissue destruction has not been understood. Although the pathological studies in periodontitis have made a significant advancement, the overall understanding of the role of immunity in the pathogenesis of periodontal diseases is still limited. The understanding of the exact role of immune cells in periodontitis is crucial for discovering novel treatment strategies.

In this review, we have summarized the latest researches describing the role of different subsets of CD4^+^ T which include helper T cells type 1 (Th1), Th2, Th17, and regulatory T (Treg) cells in the induction of immune response in chronic periodontal diseases. Moreover, we focused on the CD4^+^ T regulation by mTOR signaling, which is known to control cell differentiation, activation, and fate determination in CD4^+^ T cells. T cell activation is accompanied by a wide variety of changes in cellular metabolism and is guided by multiple cues derived from the immune microenvironment (17). The mTOR cascade is the central integrator of these signals and has an essential role in driving T cell differentiation and function (17, 18). This way mTOR pathway impacts the selection and fate of CD4^+^ T cells which ratify their role in the pathology of periodontal diseases. This study not only elucidates the relationship between immunity and periodontal diseases but also highlights the potential directions towards the development of novel therapeutic approaches.

## Pathobiology of Periodontal Diseases

Periodontitis is characterized by the inflammation caused by microorganisms in periodontal soft tissues and the gradual loss of periodontal alveolar bone (19). The progressive destruction of periodontal tissues results in tooth looseness and eventually loss of teeth resulting in tremendous social and economic burden for patients (20, 21). Periodontitis is reported to be one of the most prevalent chronic inflammatory condition which affects more than 700 million people worldwide (21).

Historically, it was believed that periodontitis is caused by specific bacterial infections and that people are unanimously susceptible to these infections and to the damage caused by them (13). Chronic inflammatory periodontal diseases are induced by imbalanced microbial communities which are presented in the form of subgingival dental plaques. Dental plaque is a typical biofilm composed of complex microbial flora (22), and the microbes in the plaque are highly ordered and embedded in an extracellular matrix. Subgingival biofilms are dental plaques that locate at the root surface of teeth or dental implants, in which the external surfaces are exposed to the gingival tissues. The transition from healthy periodontium to inflammatory periodontium is not implicated by a single type of organism. The microbial communities in periodontal diseases exhibit dysbiosis with unregulated microbial species composition and abundance, which results in a pathogenic condition (23). Three Gram-negative organisms, *Prophyromonas gingivalis*, *Treponema denticola*, and *Tannerella forsythia*, identified as “Red Complex”, were the first organism found to be associated with periodontal disease with their enriched presence in subgingival plaques within the periodontitis patients (24). Although these microorganisms are linked with the pathological condition, they are also normally present at a low level in healthy patients without periodontal diseases, suggesting that they are pathobionts rather than pathogens. Other microorganisms in periodontal tissues which are also found to be pathobionts include *Parvimonas*, *Fusobacterium*, and *Prevotella (* 25).

Nowadays, it has been accepted that the pathogenesis of periodontitis is more complex than the presence of virulent microorganisms (13). The microbes associated with periodontitis progressively destroy the periodontal tissues by producing numerous detrimental cytokines and virulence factors including exotoxins, endotoxins, fimbriae, capsule, and metabolic products (26). However, not all individuals with periodontitis-associated microbes in their gum tissues develop periodontitis, indicating a complex multifactorial etiology associated with periodontal diseases. In addition, there are intractable cases that responded poorly to the comprehensive periodontal treatment suggesting a disease susceptibility model (27). At present, it has become apparent that, except for the microorganisms, the modifiable risk factors (eg. smoking) and non-modifiable risk factors (eg. genetic inheritance and immune response) are critical etiologic agents in periodontal disease (13).

Smoking negatively affects periodontal health, which has been proved by epidemiological, clinical and *in vitro* studies (28–31). Compared with nonsmokers, smokers have presented increased susceptibility, greater severity, and faster progression of periodontitis (32, 33). Besides, smokers lose more teeth and are less likely to be successful in periodontal treatment than nonsmokers (33). It is reported that smoking reduces gingival bleeding by reducing the number of gingival blood vessels or altering the caliber of the blood vessels perfusing the gingival tissues (34). The decreased bleeding indicates an underlying disruption of the immune response, which may lead to the increased loss of attachment and alveolar bone (34, 35). More importantly, smoking cessation seems to be favorable for the periodontium, which decreases the risk for the incidence and progression of periodontitis (36, 37).

Another new discovered etiologic agent in periodontal disease is genetic inheritance. A recent meta-analysis concluded that up to a third of cases of periodontal diseases are due to the involvement of causative genetic factors and severe periodontitis shows higher heritability than moderate periodontitis (38). The specific genes which are responsible for periodontitis are not identified yet whereas the heritability of periodontitis has been found to be regulated by epigenetic mechanisms (26, 39). The heritability considered to be a relative contribution to periodontitis denotes that certain other factors might be increasing the risk, implying that the relative contribution of genetics would be moderate (38).

Many studies have reported the associations between periodontal diseases and immunocompromised systemic diseases, such as diabetes mellitus and rheumatoid arthritis (40–43). It has been generally accepted that immune system is pivotal in the etiology of periodontitis and the major cause of periodontal diseases is an imbalance between host immunity and microbial virulence (44). The individual susceptibility of the disease is regulated by the host immunity, which is also affected by environmental factors (45). Exploring the inflammatory conditions in periodontal tissues with their mode of inflammation and tissue destruction holds a pronounced significance. Understanding the regulatory pathways in inflammatory response causing periodontal tissue destruction may pave way towards a new therapeutic avenue.

Inflammation is the physiological response to the injury or the infection. In case an injury persists, the acute immune response transforms into a chronic immune response which is accompanied by the activation of adaptive immune responses. The innate and adaptive immunity must be coordinated to return the injured tissue to homeostasis, including the repair and the regeneration of lost or damaged tissues (45). Knowledge of how immunological mechanisms and inflammatory responses regulation is critical for understanding the pathogenesis of periodontitis (45). The innate immune system constitutes cells of hematopoietic and nonhematopoietic origins, including myeloid cells of hematopoietic origin (phagocytes) and epithelial cells (46). Besides these cells, there is an innate humoral response through the complement cascades. Innate immunity is a non-specific type of immunity characterized by phagocytosis in which macrophages and neutrophils digest microorganisms and foreign substances (47). When infection does not clear off, it leads to the formation of a chronic lesion, stimulating the innate immune response which eventually activates the adaptive immune response. The adaptive immune response is specific to the pathogen presentation. The cells of the adaptive immune response are lymphocytes, including B cells and T cells which are associated with antibody responses and cell-mediated immune responses, respectively.

**Figure 1:**
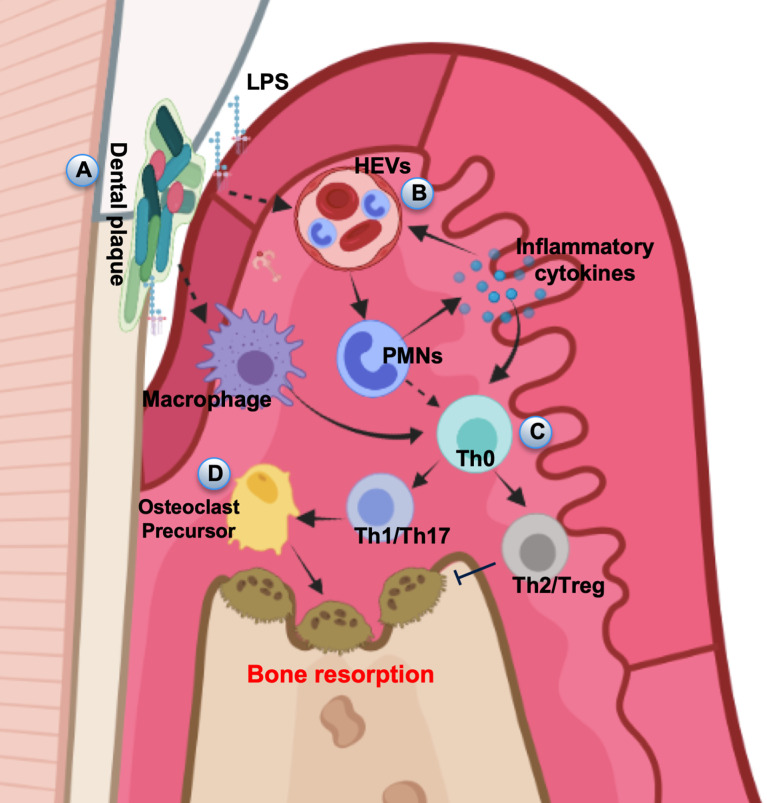
Pathogenesis of periodontal disease. **(A)** In periodontal tissue, the dental plaque stimulates local inflammatory and immune responses. **(B)** LPS and other plaque PAMPs as well as DAMPs activate the HEVs leading to vascular hyperpermeability and leakage PMN transmigration. **(C)** APCs interact with naive T helper cells, driving their differentiation into several subsets. **(D)** The amplification of local immune response leads to the development of inflammation and results in the progression of periodontal destruction and bone resorption.

In periodontal tissue, the formation of polymicrobial biofilm (plaques) stimulates a local inflammatory and immune reaction (**Figure 1A**). Currently, pathogen-associated molecular patterns (PAMPs) derived from pathogens and damage-associated molecular patterns (DAMPs) released from damaged or necrotic host cells have been considered to be crucial for inducing innate immune responses in bacterial infection (48). The recognition of PAMPs and DAMPs by host cells initiates innate immune response through toll-like receptors (TLRs) (49, 50). TLRs present on periodontal epithelial cells and immune cells can recognize highly conserved structures of bacteria, such as lipopolysaccharide (LPS), peptidoglycan, and double-stranded RNAs (51). LPS and other plaque PAMPs as well as DAMPs activate the high endothelial venules (HEVs) in the gingival lamina propria (52, 53) (**Figure 1B**). The injection of LPS from various microorganisms into the gingival tissues established periodontitis model characterized by increased infiltration of leukocytes, higher levels of proinflammatory cytokines, collagen degradation and alveolar bone resorption (54). LPS-activated endothelial cells (ECs) disrupted EC barrier leading to vascular hyperpermeability, leakage of albumin and polymorphonuclear (PMN) transmigration (55, 56). When PMNs transmigrate across the HEVs, they will be further attracted to the crevice by PAMPs and DAMPs. Enhanced accumulation of PMNs is associated with the increase of interleukin-8 (IL-8), intercellular adhesion molecule 1 (ICAM1), IL-1β, and tumor necrosis factor- α (TNF-α) expression level (57), which maintain EC activation. The vicious circle of PMN/HEV mutual activation may cause an exaggerated PMN response and a damage to the periodontal tissues (58). Therefore, the innate immune response is characterized by a dense inflammatory infiltration in the periodontal tissues, in which PMNs and macrophages are abundant immune cells.

When inflammation is persistent, macrophages can act as professional antigen-presenting cells (APCs), and stimulate CD4^+^ helper T cell responses (59). PMN can also be activated to function as accessory cells for T cell activation (60). APCs interact with naive T helper cells (Th0), driving their differentiation into several subsets, including Th cells and Treg cells (61) (**Figure 1C**). Activated lymphocytes initiate adaptive immunity and secrete proinflammatory cytokines and chemokines (62). The intensification of the local immune response leads to the expansion of inflammation and results in the progression of periodontal destruction including alveolar bone loss (63) (**Figure 1D**). Besides, memory T cells are crucial part of the immune surveillance in periodontium and important for maintaining periodontal homeostasis (64). Emphasis has been placed on the T cell response in controlling local immunity and causing chronic periodontal tissue destruction, however, minimal memory B cells also reside in the connective tissue of clinically healthy gingiva (65). The low levels of memory B cells in healthy gingiva seem to play an important role in avoiding bone loss caused by the subclinical inflammation (9). Although it is well-recognized that periodontal destruction is caused by the host inflammatory response to bacterial infection, the potential contributions and detailed molecular mechanisms of T cell differentiation and activation need to be further explored in future studies to develop effective and safe therapeutic approaches.

## The Function of CD4^+^ T Cells in Periodontal Diseases

The phenotype of periodontal diseases is characterized by the resorption of alveolar bone and tooth mobility. Adaptive immune responses have a main role in controlling the remodeling of bone (66, 67). CD4^+^ T helper cells which are an important component of the adaptive immune system, regulate the bone resorption process by producing cytokines (68). However, the development of CD4^+^ T cells is extremely complicated and strictly controlled by multiple signaling. With the stimulation by bacteria or viruses, naïve CD4^+^ T cells could differentiate into various subsets, including Th1, Th2, Th17, and Treg cells. Different subsets show distinctive function during immune response whereas the commitment of a specific subset of CD4^+^ T cells largely depends on the cytokines produced in the microenvironment. Th1 cells are characterized by the expression of interferon‐γ (IFN‐γ) (69), which are considered to mainly fight intracellular pathogens like bacteria or viruses (70). Th2 cells are defined by the expression of IL-4, IL-5, and IL-13 (71), which are perceived to mainly target extracellular parasites like helminths and allergic inflammatory responses (72). Th17 cells are defined by the expression of IL-17 and IL-22, which in responses to extracellular pathogens including bacteria and fungi (73). Treg cells exert their function by regulating immune responses to maintain immune homeostasis and prevent immunopathology (73).

In periodontitis, traditionally, Th1 cells are considered to be connected with the primary and stable periodontal lesions, whereas Th2 cells tend to be linked with progressive periodontal lesions (74). Specifically, a strong innate immune response results in the production of IL-12 by dendritic cells. The naive T cells activated by IL-12 (75) gain IFN‐γ producing capacities, which become so-called Th1 cells. The production of IFN-γ enhances the phagocytosis of both neutrophils and macrophages and hence restrain the infection (74). However, the stable lesion persists because of the continual stimulation of the dental plaque. The naive T cells can undergo a different fate and become Th2 cells when the innate response is poor and low levels of IL-12 are produced. Th2 cells produce mainly IL-4 and also produce IL-5, IL-10, and IL-13, but not IFN-γ. The stimulation of mast cells and the production of IL-4 will lead to B cells activation and antibody production. When the antibody is protective, the disease will not deteriorate. When the antibody is non-protective and cannot clear the infection, the infection will persist and the continuous activation of B cells will lead to large amounts of IL-1 and tissue destruction (74). Nevertheless, the conventional theory about Th1 versus Th2 has been proven to be unsatisfactory in the explanation of periodontal diseases since contradictory results were reported (76). In animal models of bone disease, IFN-γ, which is secreted by Th1 cells, promotes osteoclastogenesis and hence bone loss (77, 78). However, *in vitro* experiment, IFN-γ is shown to block RANKL signaling and thus inhibit osteoclasts differentiation (79), which proves a negative link between Th1 cells activation and bone resorption. These results represent the debatable role of IFN-γ and Th1 in osteoclastogenesis and bone resorption. Besides, some cytokines secreted by Th cells do not fit obviously into either category which means new Th cell subsets should be involved.

**Figure 2:**
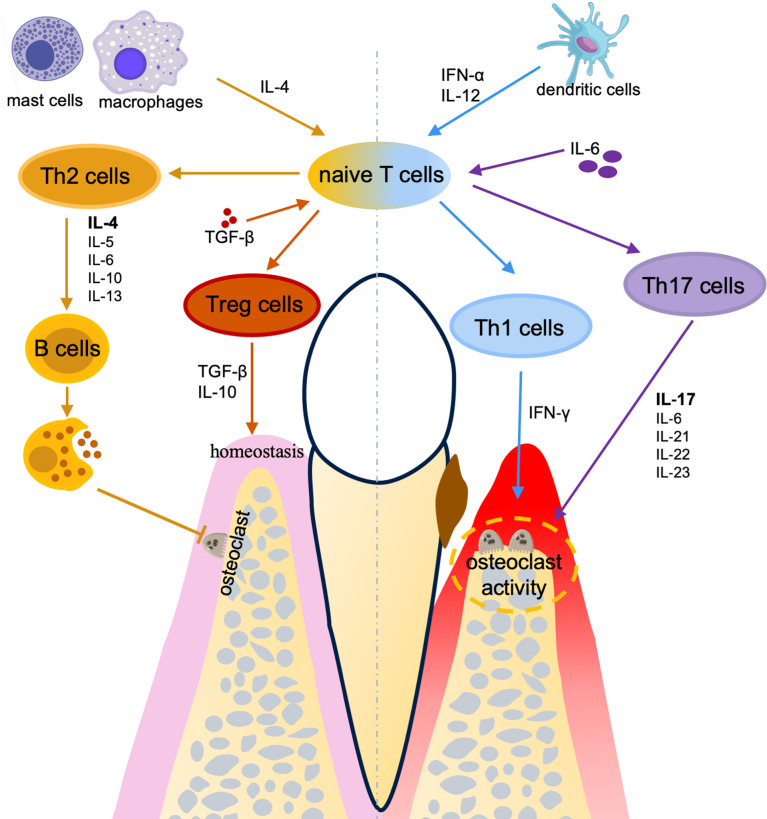
The effects of CD4^+^ T cells in periodontal diseases. A homeostasis of T helper subsets between pro-inflammation (Th1/Th17) and anti-inflammation (Th2/Treg) plays a critical role in the pathogenesis and therapies for periodontal diseases.

The role of Th1 in periodontitis is debatable, but many studies have revealed that Th1 cells are involved in the progression of periodontal diseases, especially induced osteoclastogenesis and alveolar bone loss (**Figure 2**). IFN‐γ is the signature cytokine of Th1 cells. Th1 polarization is induced by the IL-12 and IFN-α produced by the dendritic cells or the natural killer (NK) cells in the inflammatory milieu (80). Osteoprotegerin ligand (OPG-L) predominantly expresses in Th1 cells and stimulates osteoclast differentiation leading to bone resorption by the activated osteoclasts (81). The involvement of T cells in periodontal bone resorption largely depends on the recruitment of Th1 cells into the inflamed periodontal tissues (81). The injection of IFN-γ into the mice with periodontitis further enhanced the alveolar bone loss (82). Moreover, Th1 cells are found to provide pro-osteoclastogenic supports, along with pro-inflammatory cytokines that consequently lead to periodontal lesion progression (83). Interestingly, the interactions between Th cells and osteoclasts represent an intriguing aspect in osteoimmunology research field (84). In the osteoimmunology research, it is reported that T cells may promote osteoclastogenesis in an early stage of osteolysis, while subsequently osteoclasts may provide negative feedback: inhibit CD4^+^ effector T cells, block osteoclastogenesis, suppress osteoclast activity, and suppress bone resorption by FoxP3^+^ CD8^+^ T cells (85–87). Such negative feedback has also been found in periodontitis and FoxP3^+^ CD8^+^ T cells protect alveolar bone through reducing osteoclastogenesis and regulating the local immune response (88).

In contrast, Th2 cells may have protective effects on periodontal diseases by immunoregulatory and alveolar bone protection. Th2 development is induced by IL-4 (89) which is produced by the naive T cells or the mast cells/macrophages. Activated Th2 cells secrete IL-4, IL-5, IL-6, IL-10, and IL-13 to mediate the humoral immunity (90) by promoting B cell proliferation, differentiation, and antibody production, which has been proved to restrain osteoclastogenesis (91) and downregulate pro-inflammatory cytokines (45) (**Figure 2**). Cavalla et al. found the cooperation between Th2 and Treg cells which provides an anti-inflammatory and pro-reparative environment that contributes to periodontal lesion stability (83). Besides, IL-4 is the only cytokine under-expressed in periodontitis individuals but elevated after periodontal treatment (92), indicating that IL-4 and Th2 cells may have positive effects on periodontal diseases.

Despite the Th1/Th2 paradigm being a dogma to describe the development of inflammatory diseases for a long period (93), another subgroup of Th cells producing IL-17 is identified, which modified Th1/Th2 paradigm (94). Th17 cells are later found to be an essential mediator for bone destruction in periodontal diseases (95, 96). Homeostatic oral Th17 cells are commensal-independent and IL-6-dependent, whereas the development and maintenance of Th17 cell-associated periodontal diseases is largely dependent upon the local microbiomes and require a pro-inflammatory microenvironment (97). Many pro-inflammatory cytokines, including IL‐1β, IL‐6, IL‐21, IL‐22, IL‐23, and IL‐17, take part in the pathogenesis of periodontal diseases (98) (**Figure 2**). These cytokines are either stimulated or balanced by the Th17 cell-associated immune responses. Inhibiting the IL-6 signaling in dendritic cells induces a significant decrease of the Th17 cells (96). In addition, IL-17 inhibits the expression of osteoprotegerin and promotes the expression of receptor activator for nuclear factor-κB ligand (RANKL) in periodontal ligament cells, which are two essential factors for osteoclastogenesis. Thus, IL-17 may have a destructive effect on periodontal bone remodeling (99). Besides, patients with TH17 cell defects presented diminished periodontal inflammation and alveolar bone loss, despite the increased recurrence of oral fungal infections (97). It is also clear that excessive activation of Th17 leads to connective tissue destruction and bone resorption (96). Pathogenic TH17 cells in bone resorption are converted from exFoxp3^+^ T cells (100). Recently, the bone damage and tooth loss induced by exFoxp3^+^ TH17 cells are considered to be used for protecting against bacteria and stopping local infection, which means TH17 cells function as a two-edged sword by protecting against infection while inducing bone tissue resorption (100).

Treg cells play a vital role in maintaining immune homeostasis (101). Treg cells development is induced in the presence of TGF-β. Besides, in the absence of IL-6 and IL-1, the development is promoted by IL-2 and retinoic acid (RA) (93). Treg cells secrete TGF-β and IL-10 which are critical for the regulation of inflammatory responses (93) (**Figure 2**). Nan Ge et al. found that Treg cells are negatively associated with the expression levels of RANKL and the number of osteoclasts in periodontitis patients (102). It has been shown that the imbalance between Th17 and Treg cells accounts for the major pathogenesis of chronic periodontal diseases whereas Treg cells are usually considered to have a protective role (103, 104). Compared to healthy individuals, periodontitis patients have a decreased expression level of Treg cell-related gene Foxp3 and increased levels of Th17 cell-related genes RAR-related orphan receptor C (Rorc) and IL-17A (103). Moreover, the variation of the microenvironment can change the inflammatory cytokine milieu and reshape the adaptive immune response (105). Both Treg and Th17 cells can transdifferentiate into IFN-γ-producing Th1-like cells (105, 106). Collectively, homeostasis of T helper subsets between pro-inflammation (Th1/Th17) and anti-inflammation (Th2/Treg) plays a critical role in the pathogenesis and therapies for periodontal diseases. Although a certain understanding of the role of different CD4^+^ T cell subsets in periodontal diseases is achieved, further studies are still in progress for the identification of the core mechanisms that regulate the differentiation and activation of CD4^+^ T helper cells. Discovering the crucial pathways to control the differentiation of CD4^+^ T cells may bring a new direction for the treatment of periodontal diseases through regulating immunity and achieving a balance between fighting infection and reducing the tissue destruction in periodontitis.

Th22 and Th9 cells have been identified as new Th cells subsets which are phenotypically distinct from other Th cells (107, 108). Th22 cells mainly secrete IL-22 which is also produced by Th17 cells, but Th22 cells hardly produce IL-17 (109). IL-22 produced by Th22 cells are associated with alveolar bone resorption and the severity of periodontitis (110). Th9 has a potential role in tissue healing by downregulating the differentiation of Th1, Th17, and Th22 cells (111, 112). When Th9 cells were overexpressed, Th17 cells would decrease with decreased bone resorption (110). In general, the Th1/Th17/Th22 and Th2/Th9/Treg axis play antagonistic roles in periodontitis: the Th1/Th17/Th22 axis is related to the periodontal tissue destruction and alveolar bone loss during active periodontitis while the Th2/Th9/Treg axis is relevant to periodontitis remission, which is consistent with the data in other bone disorders (113).

## The Role of mTOR Signaling in CD4^+^ T Cell Differentiation and Activation

The serine/threonine protein kinase mammalian target of rapamycin (mTOR) signaling is critical for the modulation of immune responses (114). mTOR is a downstream target of the phosphatidylinositol 3-kinase-related kinase family (115), in which mTOR serves as a main component of two protein complexes, mTOR complex 1 (mTORC1) and mTORC2, exhibiting different functions and regulating different cellular processes (116). The mTORC1 is composed of mTOR, regulatory-associated protein of mTOR (Raptor), mammalian lethal with SEC13 protein 8 (mLST8, also known as GβL), PRAS40, and DEP domain-containing mTOR-interacting protein (DEPTOR) (117, 118). The mTORC2 consists of seven protein subunits: the mTOR, Rapamycin-insensitive companion of mTOR (Rictor), mammalian stress-activated protein kinase interacting protein 1 (mSIN1), protein observed with Rictor 1 and 2 (Protor1/2), DEPTOR, mLST8, and TTI1/TEL2 (119, 120). It is recognized that mTOR signaling dictates T cell fate through interaction and balance between mTORC1 and mTORC2 (121). mTOR signaling controls the function of dendritic cells (DCs), which are the antigen-presenting cells that encounter and capture oral microbes and then migrate to lymph nodes to regulate the differentiation of CD4^+^ T cells. Specifically, mTOR inhibition has suppressive effects on DC differentiation and maturation (114, 122). mTOR pathway also plays a critical role in regulating T cell activation and differentiation. The blockade of mTOR signaling leads to significant thymic involution and a decreased T-cell output (123). In addition, suppression of mTOR cascades during T cell activation also causes immunosuppression (114). These findings point out the role of mTOR signaling in regulating CD4^+^ T cell activation and differentiation for periodontal tissue homeostasis.

Both mTORC1 and mTORC2 promote Th1 cell differentiation *via* modulating cytokine signaling (124, 125). Without the mTORC1 activator Rheb (Ras homolog enriched in brain), CD4^+^ T cells are unable to secrete IFN-γ under Th1 polarizing condition (126). The mTORC1 could regulate Th1 differentiation by controlling the phosphorylation of canonical Th1 transcription factor T-bet (T-box expressed in T cells) (126). With defective mTORC2 signaling, naïve T cells show an impaired ability to differentiate into Th1 cells (125). Complementation with active Akt, an upstream kinase of mTOR complexes, can restore the expression of T-bet and thus, the Th1 cell differentiation (125). Besides, mTORC1 and mTORC2 inhibit the suppressor of cytokine signaling (SOCS) proteins (124). Rheb deficiency reduces mTORC1 activation in T cells, and Rheb-deficient T cells are unable to develop into Th1 cells (124). Knockdown of SOCS3 in Rheb-deficient T cells significantly increases Th1 cell differentiation, further indicating the direct role of mTORC1 cascade in Th1 cell selection and immune homeostasis (124).

**Figure 3:**
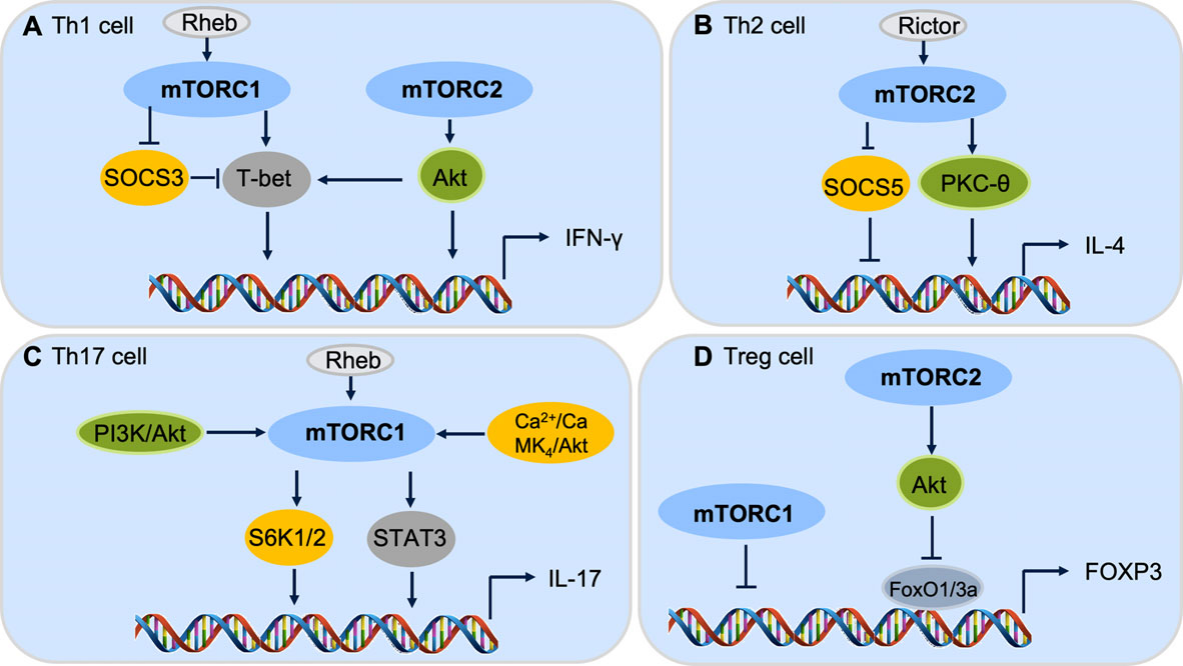
mTOR complexes control CD4^+^ T cell differentiation. **(A)** Both mTORC1 and mTORC2 pathways are involved in Th1 cell differentiation. **(B)** mTORC2, but not mTORC1, cascade regulates Th2 cell differentiation. **(C)** mTORC1 is critical for Th17 cell differentiation through downstream S6k or STAT3 signaling. **(D)** Both mTORC1 and mTORC2 cascades are important to repress Foxp3 expression and Treg cell differentiation.

mTORC2, but not mTORC1, signaling is required for Th2 cell differentiation (124, 125). Rictor is a crucial adaptor protein for mTORC2 whose deletion in CD4^+^ lineage cells leads to a deficiency in Th2 cell differentiation (124). Complementation experiments in the Rictor knockout T cells show the activation of PKC-θ *via* GATA3 (GATA binding protein 3) transcription factor and restoration of Th2 differentiation (127). Similarly, the knockdown of SOCS5 in Rictor-deficient T cells can result in an increased Th2 differention (124). As Rheb-deficient T cells retain the ability to generate Th2 cells (124), consequently, both mTORC1 and mTORC2 may involve in Th1 differentiation, while mTORC2 is required to maintain the Th2 cell homeostasis (**Figures 3A, B**).

mTORC1 is primary signaling required for Th17 cell differentiation. The PI3K-Akt-mTORC1-S6K1/2 axis is required for inducible Th17 cell differentiation (128, 129), in which depletion of Rheb in CD4^+^ lineage cells significantly decreases the differentiation of Th17 cells (124) (**Figure 3C**). Furthermore, the mTOR-STAT3 signaling also participates in Th17 differentiation (130, 131). STAT3 activation promotes the IL-17 expression by binding to the promoter regions of the IL-17 gene which leads to histone modifications of enhancer element and the expression of IL-17 increase (131). The Ca^2+^-CaMK_4_-Akt-mTOR axis has also been reported to be involved in Th17 differentiation (132). As the PI3K-Akt-mTOR pathway regulates multiple subsets of CD4^+^ T cells, CaMK_4_ is restricted to Th17 cell differentiation, indicating that CaMK_4_ may be a specific target for Th17 homeostasis (132). Despite it being widely accepted that mTORC1 is dominant signaling to promote Th17 cell differentiation, mTORC2 may also be critical for Th17 cell differentiation under special conditions, such as without the help of IL-23 (133). While deletion of Rictor in CD4^+^ lineage cells has no influence on Th17 differentiation (125), the role of mTORC2 cascade in Th17 cell differentiation is still controversial.

For Treg cells, both mTORC1 and mTORC2 show inhibitory effects on the induction of Treg cells through different downstream mechanisms (121, 134). It has been reported that disruption of mTORC1 signaling results in preferential differentiation of naive T cells into Treg cells (121). The PI3K-PDK1-Akt signaling activates mTORC1 activity through inhibiting the Tsc1/Tsc2 repressor complex, leading to the activation of mTORC1 downstream phosphorylation of S6K and 4E-BP1 to regulate Treg cell proliferation and differentiation (124, 134). Moreover, mTORC2-Akt-FoxO1/3a signaling inhibits the differentiation of Treg cells (135). These data reveal the roles of mTORC1 and mTORC2 in Treg cell differentiation *via* distinctive regulatory mechanisms (**Figure 3D**).

In summary, Th1 and Th17 cells are mainly considered to be associated with periodontal lesion progression and alveolar bone resorption, while Th2 and Treg cells are found to have roles in immunoregulatory and alveolar bone protection. Since mTOR signaling could promote Th1 and Th17 cell differentiation and inhibit Treg commitment through different mTOR complexes, we anticipate a disadvantageous effect of mTOR signaling on periodontal diseases though regulating CD4^+^ T cell subsets, though the influence of mTOR signaling on Th22 and Th9 is not clear yet.

## The Potential Roles of mTOR Signaling in the Pathogenesis of Periodontal Diseases

**Figure 4:**
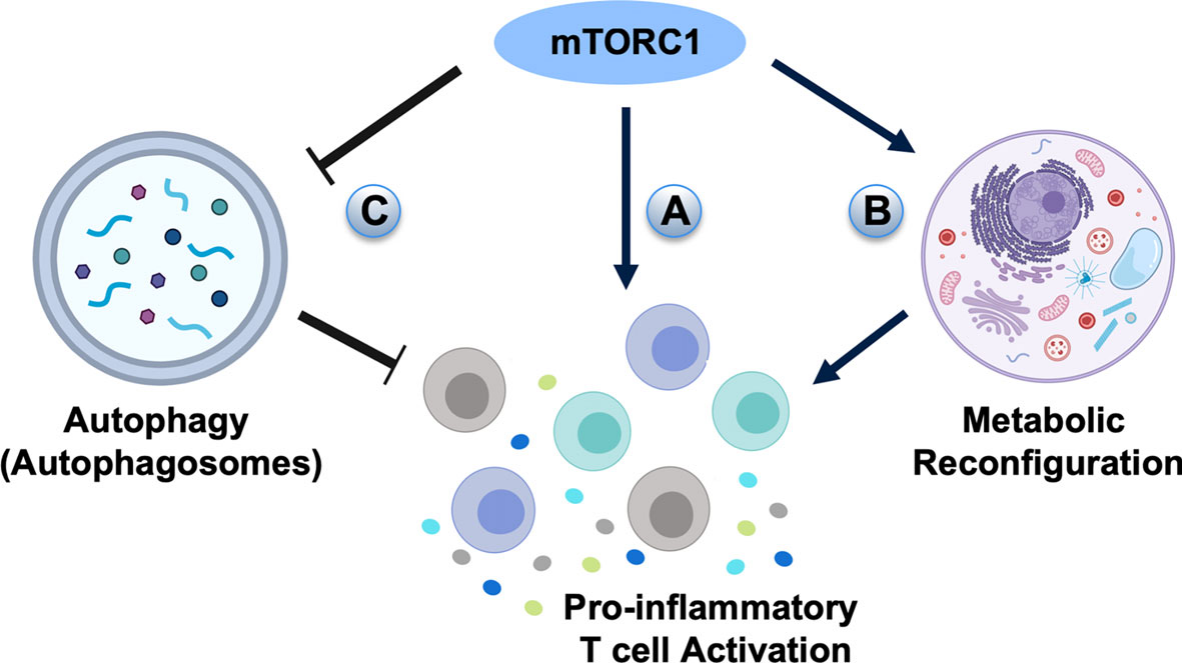
The potential roles of mTOR signaling in the pathogenesis of periodontal diseases. **(A)** mTORC1 signaling may directly activate pro-inflammatory T cell differentiation in periodontal diseases. **(B)** High mTORC1 activity elevates glycolytic flux and generates T cell populations with enhanced effector capacity. **(C)** As autophagy is inhibited by mTORC1, the negative effect of mTOR signaling on periodontitis may go through autophagic regulation.

Several studies have revealed that mTOR signaling has an adverse influence on aging-related periodontal disease (136, 137). The elderly have increased susceptibility to periodontal diseases and the aging periodontal tissue tends to react violently to periodontal pathogens (138, 139). Aging might cause increased susceptibility to periodontitis through alteration of inflammatory status and innate immunity of the host (139), but the clinical symptoms, pathological changes and pathogenic factors in aging periodontitis are similar to general periodontitis. The inhibition of mTOR signaling could relieve inflammation by down-regulating the expression levels of IL-6 and IL-8 in the aging periodontium (136). Additionally, the inhibition of mTOR with rapamycin treatment has been reported to prevent or reverse age-associated alveolar bone loss (137). Activation of mTOR signaling by ethanol stimulation could suppress human dental pulp cell differentiation and mineralization (140), while rapamycin-induced inhibition of mTOR signaling significantly diminishes odontoblastic differentiation and mineralization (140). However, whether mTOR signaling is involved in periodontitis through regulating CD4^+^ T cell differentiation remains unknown. Given the extensive roles of mTOR signaling in regulating CD4^+^ T subset differentiation, further studies are required to reveal the role of mTOR cascades in the pathogenesis of periodontitis, which may provide a novel therapeutic avenue to treat periodontitis that is currently lacking effective treatment procedures in the clinic (**Figure 4A**). There are medicines targeting the mTOR-signaling which have already been used in clinical for treating cancers (141, 142) and may be beneficial for the treatment of periodontitis in the future.

In addition to directly guiding T cell differentiation, mTOR signaling also has an important role in regulating cell metabolic pathways, including glycolysis, fatty acid synthesis, and inhibition of autophagy (143, 144). mTORC1 and mTORC2 generally promote the anabolic response, such as stimulating glycolysis, protein synthesis, and lipid metabolism to influence T cell proliferation and survival, as well as hematopoietic stem cell maintenance and differentiation (143). Deactivation of the mTOR pathway maintains T cell homeostasis under immune activation by optimizing antigen presentation and memory T cell generation (143). The asymmetric inheritance of mTOR cascades influences naive CD8^+^ T cell glycolytic metabolic capacity and cell fate determination (145). T cells with high mTORC1 activity are shown to have raised glycolytic flux and generate T cell populations with augmented effector capacity, whereas T cells with lower mTORC1 activity exhibit increased lipid metabolism and generate long-lived memory T cells (146) (**Figure 4B**). Although the role of CD8^+^ T cells in periodontitis is less obvious, CD8^+^ T cells of gingival tissues show regulatory/suppressor properties, which are critical for the gingival tissue integrity as they initiate tissue repair mechanisms under injuries and down-regulate inflammation for tissue homeostasis (147).

Autophagy is a highly controlled biological process characterized by the degradation of cellular organelles, cytoplasm, lipids, or proteins under nutrient deprivation or stressed situations (148). The mTOR signaling, specifically mTORC1, is directly involved in the formation of autophagic vesicles (144). When nutrients are abundant, the mTORC1 phosphorylates inhibitory sites on the Unc-51 like kinase-1 (ULK1) and the adapter protein autophagy-related gene-13 (Atg13), thus restraining the induction of autophagy. In starvation, mTORC1 dissociates from the ULK1 and releases ULK1 to directly phosphorylate Atg13 for the induction of autophagy (148, 149). Deletion of Atg7, a critical autophagic gene, in mature circulating T cells leads to survival defect (150). Furthermore, after TCR stimulation, the Atg5-deleted T cells are not able to proliferate and undergo apoptosis (151), indicating that autophagy is indispensable for the survival and active homeostasis of resting immunological naive T cells (150), in which Th2 cells show a higher level of autophagy than Th1 cells *in vitro* (152). Since autophagy is inhibited by mTORC1, the ability of Rheb-deficient CD4^+^ T cells to differentiate into Th2 cells might be promoted by the ability to utilize autophagy (17). More importantly, autophagy is critical in the pathogenesis and progress of periodontitis, in which periodontal pathogen invasion is controlled by autophagy. Autophagy can act through inactivation and elimination of intracellular pathogens (153), whereas periodontal pathogens such as *P. gingivalis* can induce autophagy (154). It is generally believed that autophagy is a negative controller of inflammasome activation (155–157) and has a protective role on periodontal tissues (158). As autophagy is inhibited by mTORC1, the negative effect of mTOR signaling on periodontitis may go through autophagic regulation (**Figure 4C**). The exact roles of mTOR-autophagy cascades on the initiation and progression of periodontitis still need to be evaluated through further studies.

## Prospective

Periodontal disease is triggered by the microorganisms and the resulting activation of the host immune responses (159). The immune responses which are responsible for protection against infection can also cause periodontal tissue destruction (11). Adapting the local host immune responses, regulated by CD4^+^ T cells, is a potential mechanism for interfering with the pathogenesis of periodontal diseases. Strategies for regulating CD4^+^ T cell differentiation and activation might be the potential therapeutic targets for periodontal bone regeneration. This may include the regulation of mTOR signaling, which is crucial for CD4^+^ T cell differentiation. Since different mTOR complexes play distinctive roles on CD4^+^ T cell fate determination, explicit deletion or activation of mTORC1 and mTORC2 has been recommended to explore their exact roles on periodontitis. Current studies exploring the influences of mTOR signaling on aging periodontium do not distinguish the roles of mTORC1 and mTORC2, nor do they explain the possible mTOR downstream targets, such as metabolic pathways and the autophagic regulations to control T cell differentiation (136, 137). Thus, further studies are necessary for this field to find the underlying mechanisms of CD4^+^ T cell-mediated immune responses. In addition, novel targets are required to be explored for regulating immunological networks in periodontal diseases. It will provide a better understanding of the disease and the development of novel therapeutic strategies for periodontal tissue regeneration and periodontitis treatment.

## Author Contributions

Conceptualization, CC, QJ, and XH. Writing – Original Draft Preparation, QJ, XH, WY, and CC. Writing – Review & Editing, QJ, XH, WY, RH, XZ, and CC. All authors contributed to the article and approved the submitted version.

## Funding

This work was supported by grants from National Institute of Dental and Craniofacial Research, National Institutes of Health, Department of Health and Human Services (R00DE025915, R03DE028026, and R01DE027901 to CC), and a Colgate Palmolive Grant (A-2019-590-OC) to CC.

## Conflict of Interest

The authors declare that the research was conducted in the absence of any commercial or financial relationships that could be construed as a potential conflict of interest.

## Publisher’s Note

All claims expressed in this article are solely those of the authors and do not necessarily represent those of their affiliated organizations, or those of the publisher, the editors and the reviewers. Any product that may be evaluated in this article, or claim that may be made by its manufacturer, is not guaranteed or endorsed by the publisher.
